# Catastrophic antiphospholipid syndrome with eosinophilia mimicking hypereosinophilic syndromes with disseminated intravascular coagulation: a case report

**DOI:** 10.1186/s13256-026-05851-0

**Published:** 2026-02-03

**Authors:** Hikari Ota, Tomoyuki Yoshizaki, Satoshi Nakayama, Rintaro Wakamiya, Ayano Matsunaga, Hiroaki Takeo, Kazuhiro Masuoka

**Affiliations:** 1 Department of Hematology, Mishuku Hospital, 5-33-12 Kamimeguro, Meguro-ku, Tokyo, 153-0051 Japan; 2 Department of Endocrinology and Metabolism, Mishuku Hospital, Tokyo, Japan; 3 Department of Gastroenterology, Mishuku Hospital, Tokyo, Japan; 4 Department of Pathology, Japan Self-Defense Forces Central Hospital, Tokyo, Japan

**Keywords:** Antiphospholipid syndrome, Catastrophic antiphospholipid syndrome, Eosinophilia, Hypereosinophilic syndromes, Disseminated intravascular coagulation

## Abstract

**Background:**

Catastrophic antiphospholipid syndrome is a rare variant of antiphospholipid syndrome where the presence of antiphospholipid antibodies and systemic inflammation leads to the rapid onset of multifocal thrombosis. Eosinophilia is known to accompany conditions such as allergy, parasite infection, malignancy, or autoimmune diseases; however, catastrophic antiphospholipid syndrome with eosinophilia has not been reported and its clinical influence remains unknown. We describe the first case of probable catastrophic antiphospholipid syndrome with eosinophilia, which mimicked eosinophil-associated disorders such as hypereosinophilic syndromes or eosinophilic granulomatosis with polyangiitis with disseminated intravascular coagulation, adding a new aspect of differential diagnosis of eosinophilia.

**Case presentation:**

A 46-year-old previously healthy Japanese man presenting with fever, abdominal pain, and skin lesions with pruritus showed marked eosinophilia, thrombocytopenia, and coagulopathy. A dynamic contrast-enhanced computed tomography scan of the abdomen showed some nonenhancing lesions in both lobes of the liver and the portal vein thrombosis. Upper gastrointestinal endoscopy showed gastric erosions, and lower gastrointestinal endoscopy revealed transverse colon and cecum ulcers. Common causes of eosinophilia including allergy, infection, and medication were not detected. He was initially suspected with hypereosinophilic syndromes or vasculitis such as eosinophilic granulomatosis with polyangiitis complicated by disseminated intravascular coagulation, and was treated with prednisone and thrombomodulin from hospital day 4 after bone marrow examination and the biopsies of the skin, stomach, and colon; however, these examinations excluded neoplastic hypereosinophilic syndromes and vasculitis. Later examination revealed positive antiphospholipid antibodies including lupus anticoagulant, anticardiolipin antibodies immunoglobulin G, and anticardiolipin β2-glycoprotein 1 complex antibodies. He was complicated by colonic perforation and bilateral adrenal hemorrhage on day 10. Histopathology of the resected colon and liver biopsy confirmed arterial and venous small-vessel thrombosis and microthrombi, leading to the diagnosis of probable catastrophic antiphospholipid syndrome. He was successfully treated with plasma exchange and rituximab. He has been in remission of catastrophic antiphospholipid syndrome for 4 years, and his antiphospholipid antibodies have been negative post-rituximab treatment. His eosinophil count has been between normal to slightly increased, possibly due to the chronic adrenal insufficiency.

**Conclusion:**

Our case shows that eosinophilia can accompany catastrophic antiphospholipid syndrome, and this can mimic eosinophil-associated disorders with disseminated intravascular coagulation. Identifying antiphospholipid antibodies is important for differential diagnosis when treating unexplained eosinophilia, thrombocytopenia, and thrombosis.

## Background

Catastrophic antiphospholipid syndrome (CAPS) is a rare, devastating variant of antiphospholipid syndrome (APS) where the presence of antiphospholipid antibodies (aPLs) and systemic inflammation leads to the rapid onset of multifocal thrombosis [1]. A definitive diagnosis of CAPS requires the following four criteria: (1) involvement of three or more organs or tissues; (2) development of manifestations in less than 1 week; (3) histopathology of small vessel occlusion; and (4) presence of aPLs on two occasions 6 weeks apart [2]. If a patient has only three out of these four requirements, the patient is classified as probable CAPS [2]. Almost half of the patients who develop CAPS do not have a history of aPL positivity [3], thus such cases cannot be diagnosed with definite CAPS at onset because positive aPLs on two occasions 6 weeks apart cannot be confirmed [2]. Due to this, several previous cases of CAPS have been initially diagnosed as probable CAPS [4].

Peripheral blood eosinophilia (PBE) is known to accompany conditions such as allergy, parasite infection, malignancy, or autoimmune diseases [5]. Indeed, there have also been several reports of APS with PBE [6]; however, PBE in the setting of CAPS has not been reported and its clinical influence remains unknown. Since the condition of PBE itself can cause thrombosis and multiorgan failure [7], it would be difficult for clinicians to suspect CAPS as a potential diagnosis in the setting of thrombosis and eosinophilia rather than more common diseases such as hypereosinophilic syndromes (HES), eosinophilic granulomatosis with polyangiitis (EGPA), or occult malignancies, posing a diagnostic challenge.

Herein, we present a case of probable CAPS with persistent fever, abdominal pain, skin lesions with pruritus, PBE, thrombocytopenia, and multiple thromboses that mimicked eosinophil-associated disorders such as HES complicated by disseminated intravascular coagulation (DIC) at the time of initial assessment. Our case highlights the need for increased awareness that PBE can accompany CAPS, and the importance of identifying aPLs for the diagnosis of CAPS when treating unexplained eosinophilia, thrombocytopenia, and multiple thromboses.

## Case presentation

**Fig. 1:**
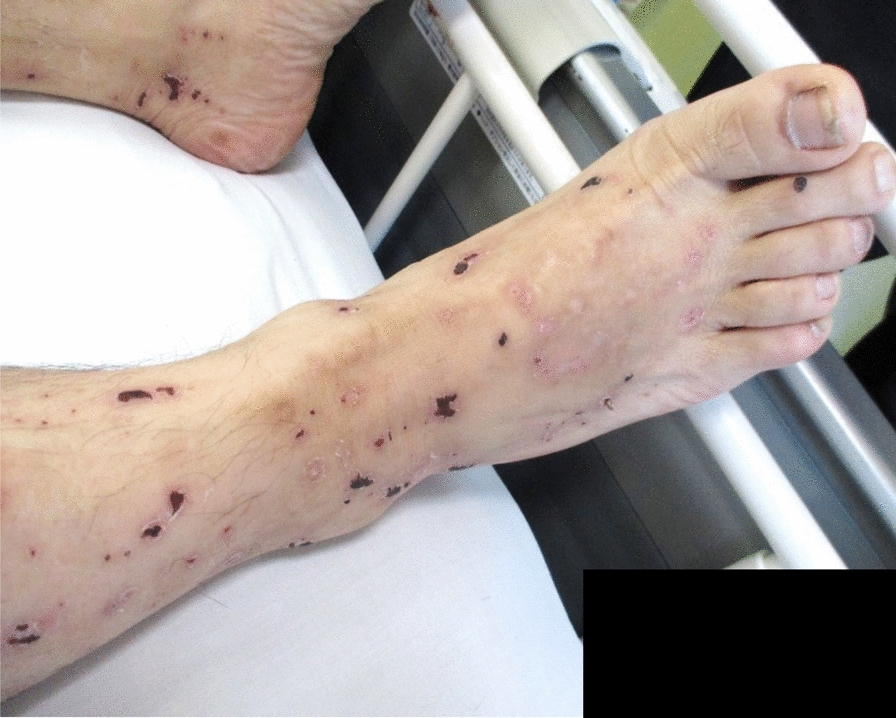
Cutaneous manifestations on admission. Multiple irregular ulcers covered with scabs, measuring approximately 2–10 mm in diameter, are distributed in an isolated pattern in lower extremities

**Fig. 2:**
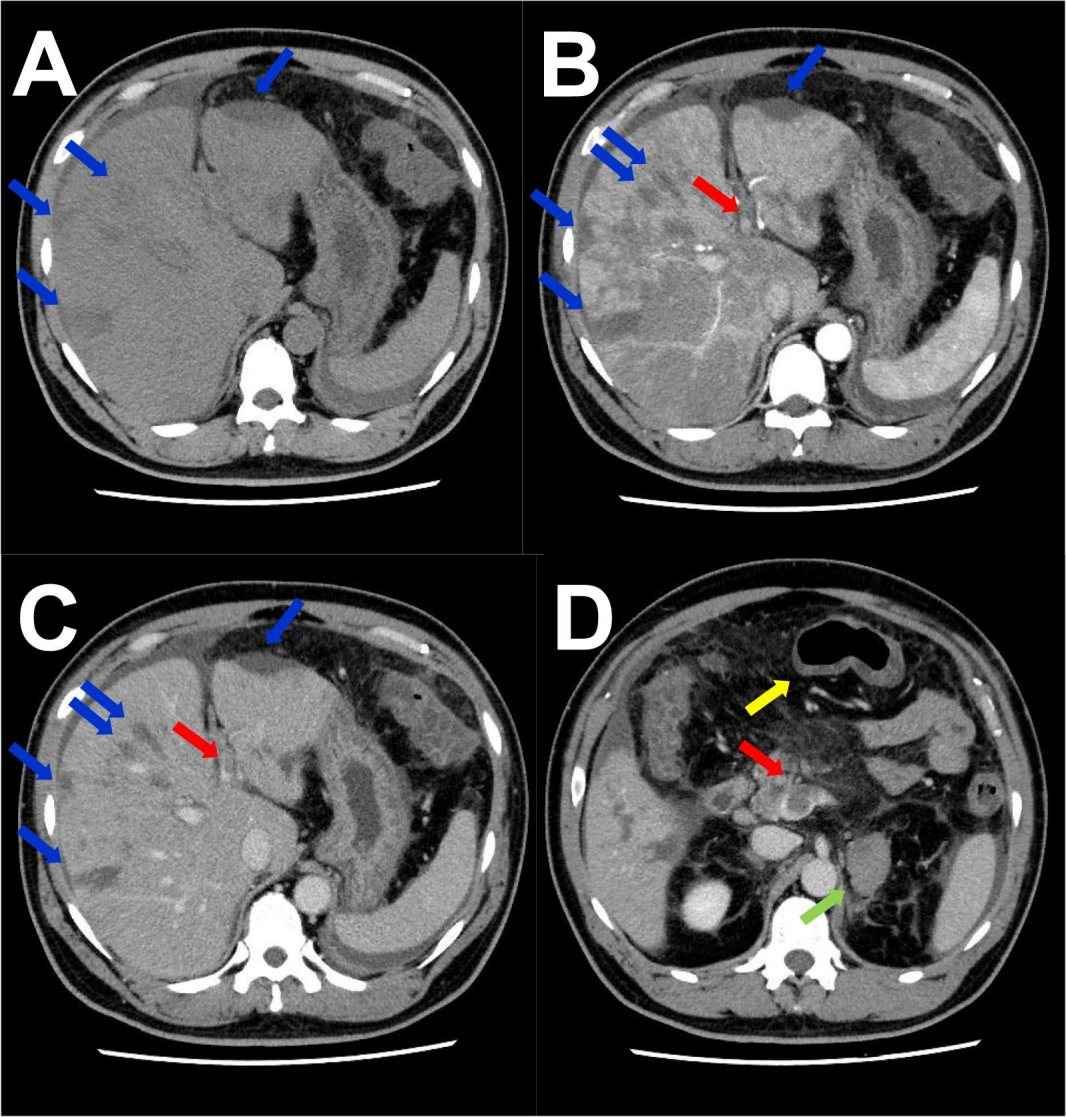
Dynamic contrast-enhanced computed tomography findings on day 2. **A** Plain computed tomography scan shows atrophy of the left lobe of the liver, some hypodense lesions in both lobes of the liver (blue arrows), and mild ascites. **B** Arterial phase of dynamic contrast-enhanced computed tomography scan shows some nonenhancing lesions in both lobes of the liver (blue arrows), enhancement in the left lobe and anterior segment of the right lobe, lack of enhancement in the posterior segment of the right lobe, and thrombosis of the left branch of the portal vein (red arrow). **C** Portal venous phase of dynamic contrast-enhanced computed tomography scan shows some nonenhancing lesions in both lobes of the liver (blue arrows) and thrombosis of the left branch of the portal vein (red arrow). **D** Enlargement of left adrenal gland (green arrow), increased wall thickness of the transverse colon (yellow arrow), diffuse increase in density of mesenteric fat tissue, and thrombosis of the main portal vein (red arrow)

**Fig. 3:**
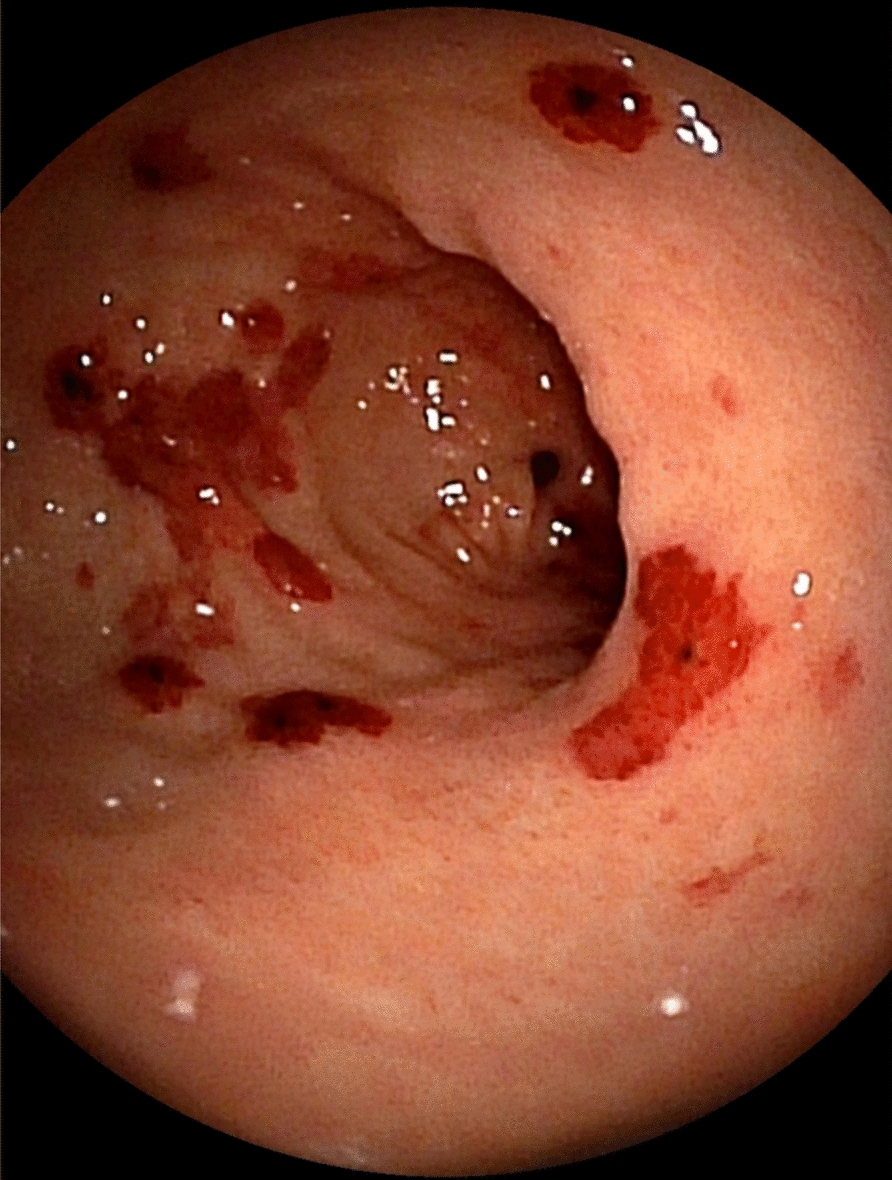
Upper gastrointestinal endoscopy findings on day 4. Multiple well-demarcated erythematous macules of various sizes are observed in the stomach

**Fig. 4:**
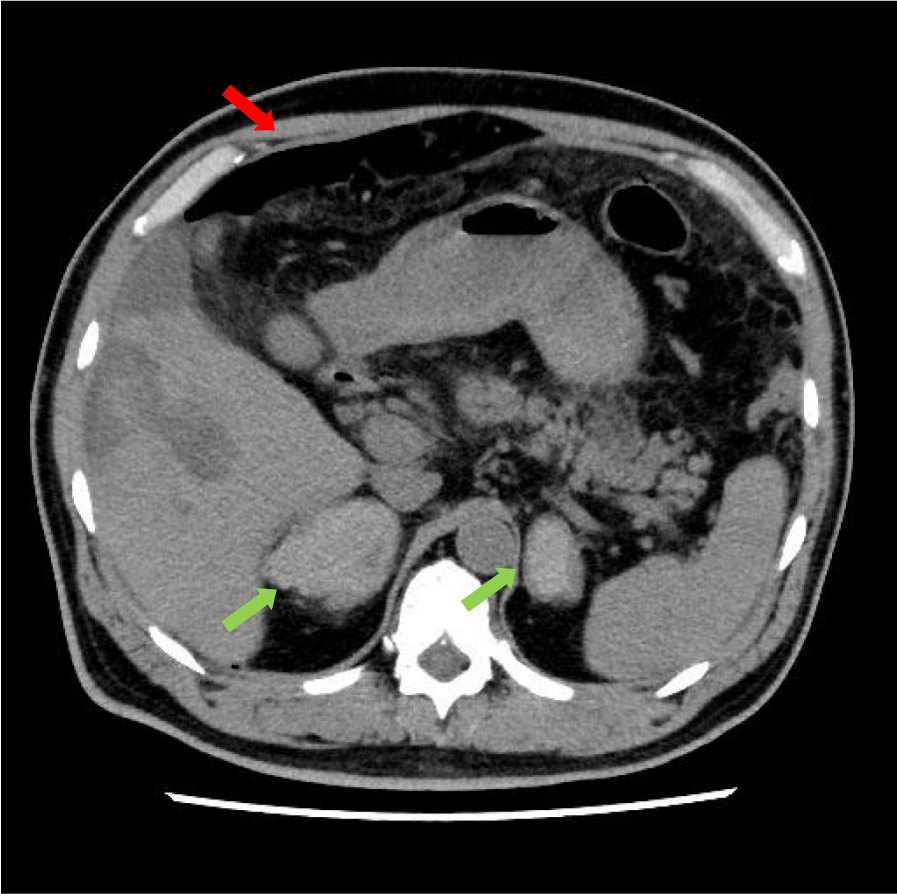
Plain computed tomography findings on day 10. Plain computed tomography scan shows some hypodense lesions in the liver, bilateral adrenal enlargement with high-attenuation (green arrows), and free air in the right side of the upper abdomen (red arrow)

**Fig. 5:**
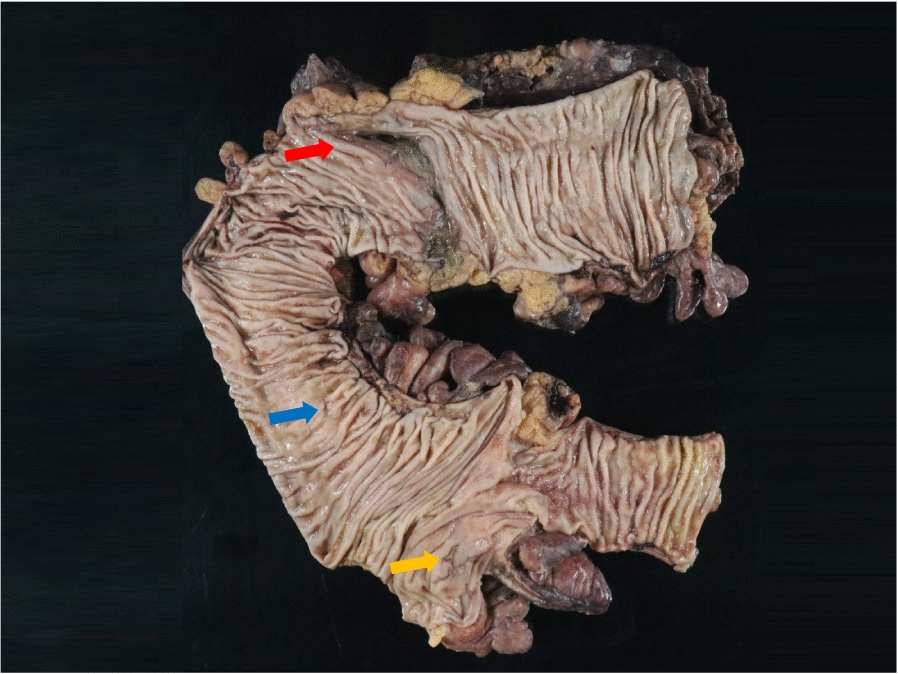
Macroscopic findings of the resected right colon. A 1.5-cm-sized ulcer is seen in the cecum (orange arrow). A 0.5-cm-sized ulcer is seen in the ascending colon (blue arrow). A whole circumference ulcer with perforation is seen in the transverse colon (red arrow)

**Fig. 6:**
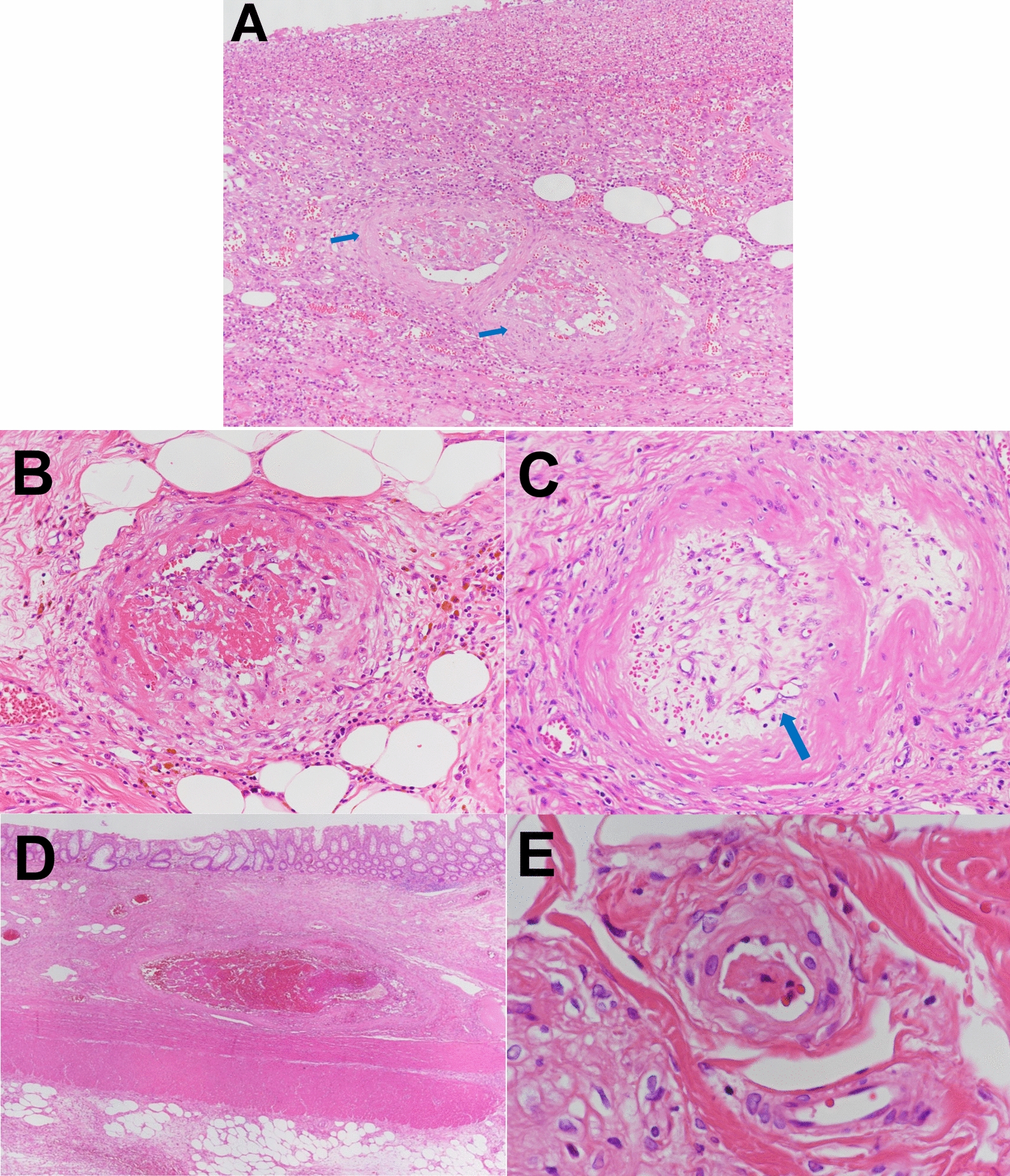
Microscopic findings of the ulcerative lesion in the colon. **A** Mucosal defect, collapse of the layer of the colon tissue with prominent neutrophil infiltration and fibrosis, and multiple thrombi in the intestinal wall are seen (blue arrows) (×100). **B**, **C** Arterial small-vessel thrombosis (×200), **D** venous small-vessel thrombosis (×20), and **E** microthrombi are seen in the intestinal wall (×600). **B** Fibrin thrombus is accompanied by a cluster of macrophages and the lysis of leukocytes and red blood cells, resulting in eosinophilic changes of the vascular wall. Hemosiderin-laden macrophages are seen around the vessel. **C** Organized thrombus with recanalization is seen (blue arrow)

**Fig. 7:**
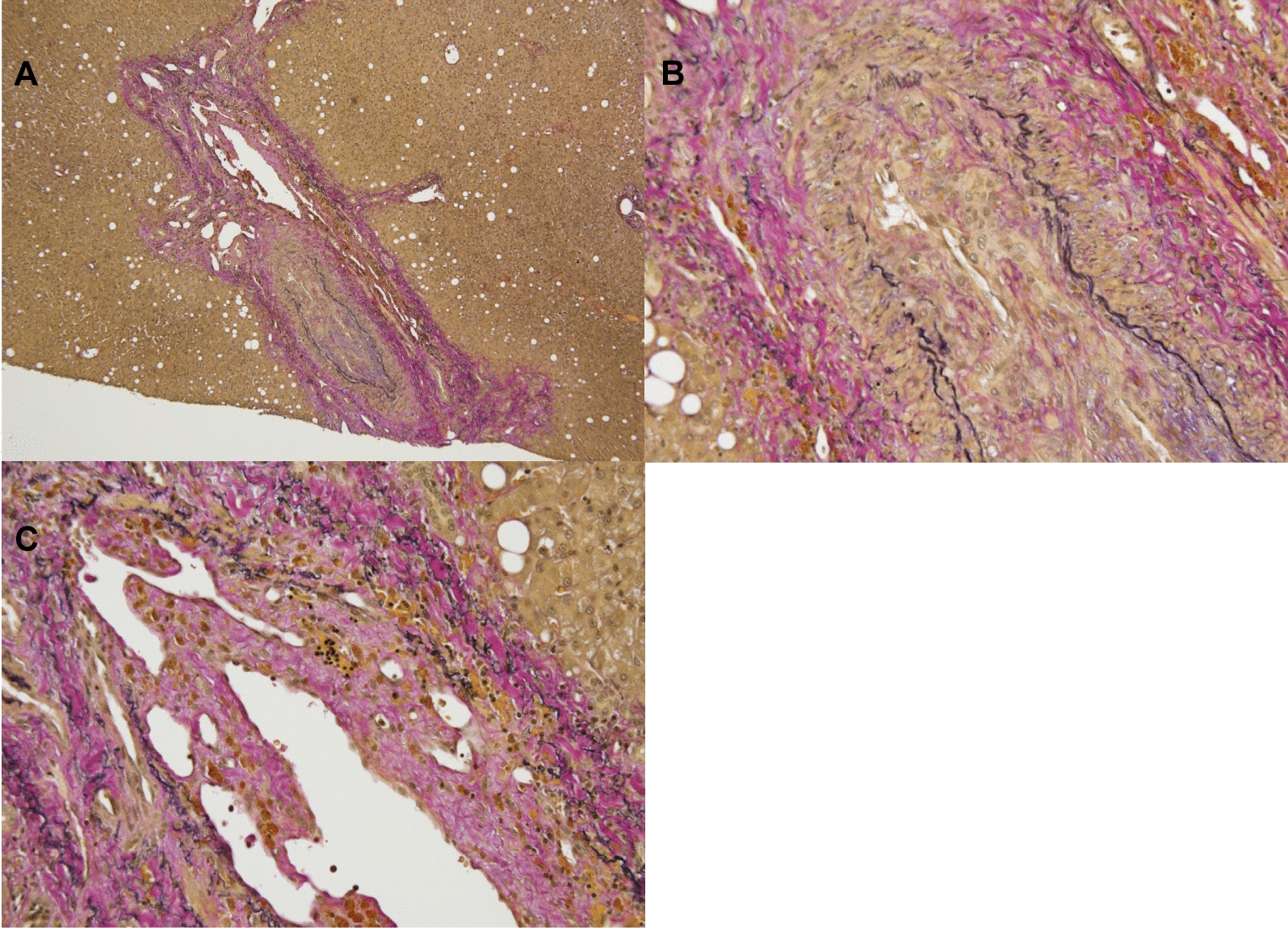
Microscopic findings of the liver biopsy specimen. **A** Organized thrombi are seen in the portal area of the liver (Elastica Van Gieson stain; ×40). **B** Organized thrombus with recanalization is seen in a hepatic arteriole (Elastica Van Gieson stain; ×200). **C** Organized thrombus with recanalization is seen in a portal venule (Elastica Van Gieson stain; ×200)

A 46-year-old Japanese man working as a security guard, with no remarkable medical or surgical history, no relevant family history, and no history of smoking, habitual alcohol consumption, recent travel, allergies, or medication use, became aware of epigastric pain after eating grilled chicken skewers and drinking beer in the evening. The pain persisted for 5 days, and he then started to have a fever and multiple palpable erythema with pruritus, and he was admitted to a hospital where he was previously treated. Laboratory data showed mild eosinophilia and liver and renal dysfunction on admission. He was treated with piperacillin/tazobactam with suspicion of a bacterial infection, but blood cultures were negative. Since a follow-up blood test performed 5 days after admission showed a further increase of eosinophils and a decrease in platelet count, he was transferred to our hospital for suspected hematological disease the next day. He complained of persistent fever, abdominal pain, and pruritus. Physical examination showed tenderness of the upper abdomen and multiple ulcerated skin lesions on the extremities and trunk (Fig. 1). Heart sounds, breath sounds, and neurological findings were unremarkable. Laboratory data (Table 1) on admission showed marked eosinophilia (white blood cell count: 21,570/µL with 32.5% eosinophils), thrombocytopenia (platelet count: 2.4 × 10^4^/µL), liver and renal dysfunction, and coagulopathy. Abdominal ultrasound showed uneven fatty liver and suggested portal vein thrombosis. Dynamic contrast-enhanced computed tomography (CT) scan of the chest and abdomen confirmed atrophy of the left lobe of the liver, some nonenhancing lesions in both lobes of the liver, thrombosis of the left branch of the portal vein and main portal vein, enlargement of the left adrenal gland, increased wall thickness of the transverse colon, and a diffuse increase in density of mesenteric fat tissue on hospital day 2 (Fig. 2). The nonenhancing lesions observed in the liver suggested impaired arterial and venous perfusion due to thrombi. Mass lesions, swelling of lymph nodes, and splenomegaly were not observed. Bone marrow examination showed hypercellular marrow with eosinophilia, with no proliferation of blast cells or dysplasia. Flow cytometry detected no specific pattern, and G-banding showed a normal karyotype. Fluorescence in situ hybridization (FISH) analysis of *FIP1L1::PDGFRA*, *PDGFRB*, *FGFR1*, and *BCR::ABL* were all negative, and T-cell receptor rearrangement analysis showed no monoclonality. These excluded hematological malignancies including T-cell lymphocytic variants of HES. At this point, some eosinophil-associated disorders including idiopathic HES and vasculitis such as EGPA complicated by DIC were suspected; thrombomodulin alfa at a dose of 380 U/kg/day was administered for the treatment of the suspected DIC and portal vein thrombosis from day 2, and further systemic examination was then implemented. Fundoscopic examination revealed soft exudates. Upper gastrointestinal endoscopy showed multiple erythematous macules in the stomach (Fig. 3), and lower gastrointestinal endoscopy under poor bowel preparation revealed ulcers of the transverse colon and cecum on day 4. Brain magnetic resonance imaging (MRI) was unremarkable. Common causes of eosinophilia including allergy, parasite infection, and medication were not detected, and malignancies were also excluded from imaging and endoscopic studies. Additional laboratory tests showed high levels of immunoglobulin E (IgE). Prednisone (PSL) 80 mg (1 mg/kg/day) was administered from day 4 for the treatment of the suspected eosinophil-associated disorders above after the biopsies of the skin, stomach, and colon. Although his fever and PBE were relieved soon after PSL administration, severe thrombocytopenia persisted. Because myeloperoxidase-antineutrophil cytoplasmic antibody (MPO-ANCA) and proteinase 3-antineutrophil cytoplasmic antibody (PR3-ANCA) were negative and histopathological results of the biopsies above showed mild infiltration of lymphocytes and eosinophils with no evidence of vasculitis and thrombosis, EGPA was also excluded. Laboratory tests performed on day 3 excluded deficiencies of protein C, protein S, and antithrombin Ⅲ and thrombotic thrombocytopenic purpura (TTP) as potential causes of thromboses, however, it revealed positivity for lupus anticoagulant (LA), anticardiolipin antibodies (aCL) IgG (40 U/mL; reference range, < 10), aCL IgM (13 U/mL; reference range, < 8), and anticardiolipin β2-glycoprotein 1 complex antibodies (aCL-β2GPI) (9.5 U/mL; reference range, < 3.5), thus he was clinically diagnosed with CAPS on day 8. We initiated PE on day 9 and his thrombocytopenia resolved the next day. However, he complained of further abdominal pain, and plain CT scan on day 10 showed free air and bilateral adrenal enlargement with high attenuation (Fig. 4), strongly suggesting intestinal perforation and bilateral adrenal hemorrhage; he underwent emergent abdominal surgery. A 55-cm-sized resected right colon had a perforated circumferential ulcer in the transverse colon and small ulcers in the ascending colon and cecum (Fig. 5). Histopathology showed a mucosal defect, collapse of the layer with prominent neutrophil infiltration and fibrosis, and multiple thrombi in the intestinal wall (Fig. 6A). Arterial and venous small-vessel thrombosis and microthrombi were observed in the intestinal wall (Fig. 6B–E), thus the ulcerative lesions were concluded to be caused by ischemia due to the thrombi. Some fibrin thrombi were accompanied by a cluster of macrophages, lysis of leukocytes, and red blood cells (Fig. 6B), and organized thrombi with recanalization were also seen (Fig. 6C). These findings indicated the thrombi had been repeatedly formed over a long period. The biopsy of the left lateral liver performed during the operation also showed organized thrombosis of hepatic arteriole and portal venule (Fig. 7), implying a preceding chronic history of APS. He was diagnosed with a probable CAPS, fulfilling the first three of the four CAPS criteria described above. The last criterion requiring the presence of aPLs on two occasions 6 weeks apart was not met. His PBE was considered as reactive against CAPS, thus idiopathic HES was excluded.

**Table 1:** Laboratory data

Complete blood cell count		(Normal range)	Biochemistry		(Normal range)	Immunologic test		(Normal range)
Red blood cell	393 × 10^4^/µL	(410–530)	Total protein	6.0 g/dL	(6.8–8.2)	IgG	1462 mg/dL	(870–1700)
Hemoglobin	12.1 g/dL	(13–17)	Albumin	2.3 g/dL	(4–5)	IgA	232 mg/dL	(110–410)
White blood cell	21,570/µL	(4000–9000)	Total bilirubin	2.4 mg/dL	(0.2–1.0)	IgM	125 mg/dL	(35–220)
Metamyelocyte	0.5%		Direct bilirubin	1.6 mg/dL	(0.1–0.3)	IgE	1320 mg/dL	(< 173)
Stab cell	0.5%	(1–7)	AST	71 U/L	(8–38)	CH50	44 U/mL	(30–50)
Segmented cell	59.5%	(40–70)	ALT	319 U/L	(4–44)	C3	60 mg/dL	(65–135)
Eosinophil	32.5%	(1–6)	LD	634 U/L	(106–211)	C4	12 mg/dL	(13–35)
Lymphocyte	5.5%	(20–50)	ALP	682 U/L	(104–338)	ANA	< × 40	(< × 40)
Monocyte	1.5%	(2–10)	Urea nitrogen	14.2 mg/dL	(8–20)	PR3-ANCA	< 1.0 U/mL	(< 3.5)
Platelet	2.4 × 10^4^/µL	(12–35)	Creatinine	1.23 mg/dL	(0.6–1.1)	MPO-ANCA	< 1.0 U/mL	(< 3.5)
Reticulocyte	1.52%	(0.5–2)	Sodium	138 mmol/L	(135–145)	LA (dRVVT)	1.52	(< 1.3)
FRC	0.1%	(< 0.5)	Potassium	3.5 mmol/L	(3.5–5)	aCL IgG	40 U/mL	(< 10)
Coagulation			Chloride	99 mmol/L	(96–106)	aCL IgM	13 U/mL	(< 8)
PT	68%	(70–130)	Glucose	192 mg/dL		aCL β2GPI	9.5 U/mL	(< 3.5)
PT-INR	1.23	(0.85–1.10)	Creatine kinase	94 U/L	(56–244)	Direct Coombs test	–	(-)
APTT	31.1 sec	(25–39)	CRP	37.2 mg/dL	(< 0.3)	ADAMTS13	30%	(> 10)
Fibrinogen	614 mg/dL	(150–350)	Ferritin	1320 mg/dL	(39.4–340)	ADAMTS13 inhibitor	− BU/mL	(–)
FDP	47.0 μg/mL	(0–5)	Haptoglobin	109 mg/dL	(66–218)	Infection test		
D-dimer	29.6 μg/mL	(0–1)	sIL2R	2860 U/mL	(145–519)	HBs-Ag	–	(−)
Protein C	59%	(64–146)	Urine test			HCV-Ab	–	(−)
Protein S	92%	(67–164)	Protein	+		HIV-Ab	–	(−)
Antithrombin	55%	(79–121)	Sugar	–		RPR	–	(−)
			Occult blood	–		SARS-CoV-2 PCR	–	(−)
			Granular cast	–		Blood culture	–	(−)

*FRC* fragmentated red blood cells, *PT* prothrombin time, *PT-INR* prothrombin time-international normalized ratio, *APTT* activated partial thromboplastin time, *FDP* fibrinogen degradation products, *AST* aspartate aminotransferase, *ALT* alanine aminotransferase, *LD* lactate dehydrogenase, *ALP* alkaline phosphatase, *CRP* C-reactive protein, *sIL2R* soluble interleukin-2 receptor, *Ig* immunoglobulin, *ANA* antinuclear antibody, *PR3-ANCA* proteinase 3-antineutrophil cytoplasmic antibody, *MPO-ANCA* myeloperoxidase-antineutrophil cytoplasmic antibody, *LA* lupus anticoagulant, *dRVVT* dilute Russell’s viper venom time, *aCL* anticardiolipin antibody, *aCL-β2GPI* anticardiolipin β2-glycoprotein I complex antibody, *HBs-Ag* hepatitis B surface antigen, *HCV* hepatitis C virus antibody, *HIV-Ab* human immunodeficiency virus antibody, *RPR* rapid plasma reagin test, *PCR* polymerase chain reaction

**Fig. 8:**
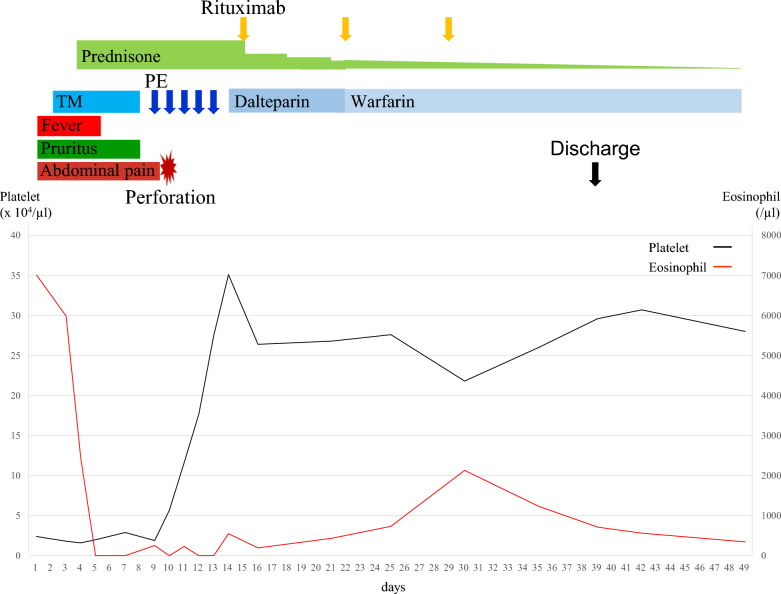
Summary of the clinical course from admission until hospital discharge. Changes in platelet and absolute eosinophil counts associated with clinical events and treatment are shown. *TM* thrombomodulin alfa, *PE* plasma exchange

Plasma exchange (PE) was performed from day 9 to day 13, and his clinical course after the operation was uneventful. To promote remission of CAPS earlier and spare high doses of PSL, weekly rituximab (RTX) 375 mg/m^2^ was added from day 15. PSL was then tapered as follows: 40 mg/day for 3 days, 30 mg/day for 3 days, from 20 mg/day at the rate of 5 mg every week, from 10 mg/day at the rate of 2.5 mg every month, from 5 mg/day at the rate of 1 mg every month, and from 3 mg/day at the rate of 0.5 mg every 2 months. In terms of anticoagulation, intravenous dalteparin at a dose of 75 U/kg/day was administered from day 14 and was switched to warfarin on day 22, with a target international normalized ratio (INR) of 2.0–3.0. His aPLs became negative on day 33. After three courses of weekly RTX, he was discharged on day 39. The clinical course during the acute phase is summarized in Fig. 8.

The follow-up CT scan showed a decrease in bilateral adrenal gland size and its change into low density, consistent with a clinical course of bilateral adrenal hemorrhage. PSL 2.5 mg was replaced with low-dose hydrocortisone for the treatment of chronic adrenal insufficiency, which was diagnosed by a rapid adrenocorticotropic hormone stimulation test, at 8 months post-treatment. He has been in remission of CAPS and his aPLs including LA, aCL, and aCL-β2GPI have now been negative for 4 years. Following the remission of CAPS, warfarin has been continued with a reduced target INR of 1.5–2.0. During the follow-up period, the patient has reported feeling well, with no skin rashes or pruritus. His absolute eosinophil count during follow-up has ranged from normal to slightly increased (median 383/µL, range 40–960/µL), possibly due to the chronic adrenal insufficiency.

## Discussion

We presented a case of probable CAPS with persistent fever, abdominal pain, skin lesions with pruritus, PBE, thrombocytopenia, and multiple thromboses, and aPLs. Our case shows that (1) PBE can accompany CAPS; (2) PBE in the setting of CAPS may cause highly aggressive thrombosis; (3) this can mimic HES with DIC at the time of initial assessment and examining aPLs is important for differential diagnosis; and (4) immediate, intensive therapy including anticoagulation, glucocorticoids, PE, and RTX is effective.

We presented a case of probable CAPS with PBE. Regarding PBE accompanying CAPS, its frequency remains unknown, and this is the first reported case of CAPS with PBE to the best of our knowledge. The descriptive analysis of 500 patients from the international CAPS Registry showed that laboratory features include thrombocytopenia (67%), schistocytes (22%), thrombotic microangiopathy (14%), disseminated intravascular coagulation (11%), lupus anticoagulant (83%), aCL IgG (81%), aβ2GPI IgG (78%), and antinuclear antibodies (57%) [8]. Because our case presented with skin lesions, pruritus, PBE, elevated IgE levels, and bilateral adrenal hemorrhage, a possible explanation of PBE shown in our case is the multifactorial nature of autoimmune disease itself, type 2 inflammation, and adrenal insufficiency. To begin with, autoimmune disorders can be associated with PBE to a various extent where eosinophils are present as part of the overall inflammatory condition [9]. Second, PBE and elevation of IgE levels are widely used as a hallmark of type 2 inflammation [10]. Additionally, in most skin disorders that cause pruritus, type 2 inflammation is considered to play a key role in pruritus induction [11]. Although our case did not have typical allergic diseases such as atopic dermatitis or asthma, some etiology of allergy and immune dysregulation might have caused PBE and elevation of IgE levels. Third, adrenal insufficiency is often associated with PBE, which is considered to be due to the loss of endogenous glucocorticoids [12]. There has also been a case report of adrenal insufficiency caused by bilateral adrenal hemorrhage accompanying APS [13]. Although our case had not been diagnosed with adrenal insufficiency at the time of initial assessment, relative adrenal insufficiency due to thrombosis can be reasonably suspected from the bilateral adrenal hemorrhage.

Second, our case of probable CAPS with PBE developed multiple thromboses within a short period of time. In general, PBE can independently be associated with thrombosis [7, 14]. Activated eosinophils trigger the coagulation cascade by causing endothelial damage due to the release of cytotoxic granules, such as major basic protein or eosinophil peroxidase, or by the expression of tissue factor, with interactions with platelets [15]. CAPS and APS are thrombotic diseases caused by aPLs, and the intensity and extent of the thrombotic process attributable to systemic inflammation is stronger in CAPS than in APS [16]. Although the precise role of eosinophils in CAPS and APS remains unknown, the coexistence of aPLs and PBE is reported to enhance the risk of thrombosis [6]. Given this evidence, our case suggests that CAPS with PBE can cause more aggressive thrombosis.

Third, our case of probable CAPS with PBE mimicked HES complicated by DIC at the time of initial assessment. HES is a heterogeneous group of disorders with sustained eosinophilia and associated organ damage, and the diagnosis of idiopathic HES is established on the basis of the exclusion of reactive eosinophilia, lymphocyte-variant of HES, chronic eosinophilic leukemia, and myeloid/lymphoid neoplasms with eosinophilia and tyrosine kinase fusion genes [17]. DIC, which is a secondary manifestation of an underlying disease characterized by excessive activation of coagulation resulting in both thrombosis and hemorrhage, can complicate HES [18, 19]. Since eosinophil-associated disorders with DIC present with thrombocytopenia and thrombosis, this can mislead clinicians to an inappropriate diagnosis, as shown in our case. Because the diagnosis of CAPS is generally difficult for clinicians due to its rarity and rapid progression, especially in idiopathic cases without a history of APS, we speculate that there can be cases of overlooked or unreported CAPS with PBE whose initial diagnosis is HES complicated by DIC, resulting in early death without adequate assessment. In such cases, a careful and timely differential diagnosis is needed. In the present case, for example, the rapidly progressing multiple skin ulcers, erythematous gastric lesions, and colon ulcers suggested systemic arterial thrombosis. Given the concomitant portal vein thrombosis, nonenhancing hepatic lesions, and adrenal enlargement possibly consistent with adrenal hemorrhage, CAPS was the leading differential diagnosis. Further, EGPA was less likely considering the lack of a history of asthma, otolaryngologic symptoms, neuropathy, or cardiac involvement. HES was also unlikely because the concomitant occurrence of arterial thrombosis in noncardiac organs and venous thrombosis is very rare in patients with HES [20]. Furthermore, acute DIC was considered atypical because there was no prolongation of activated partial thromboplastin time, no reduction in fibrinogen levels, and only a mild prolongation of prothrombin time despite the marked increase of fibrinogen degradation products and D-dimer level. In general, assessment of aPLs is recommended for cases that show thrombocytopenia and thrombosis [21]. Similar to our case, the other reported cases of CAPS also frequently showed thrombocytopenia and thrombosis (91%) [4]. Examining aPLs is important to differentiate CAPS with PBE from HES or other eosinophil-associated disorders complicated by DIC when treating unexplained eosinophilia, thrombocytopenia, and thrombosis.

The recommended treatment for CAPS is a combination of anticoagulation, glucocorticoids, and PE or intravenous immunoglobulin, a regimen referred to as “triple therapy” [22, 23]. Although the exact mechanism by which PE has an effect on CAPS is unclear, the elimination of pathogenic autoantibodies, cytokines, and complement factors are thought to ameliorate the disease [23]. Intravenous immunoglobulin is also incorporated as the third component of “triple therapy” when PE is unavailable or not effective, and is thought to exert immunomodulatory effects through a variety of mechanisms [16]. In addition, RTX, a monoclonal antibody targeting CD20 that is used to treat B-cell lymphomas, has been used to treat B cell-mediated autoimmune diseases including refractory CAPS [24], and there are reports of CAPS where aPLs became negative post-RTX treatment [24]. Because RTX is well tolerated and effective in eliminating autoantibodies, it can also be used for the initial treatment of autoimmune diseases to spare glucocorticoid use [25]. Although evidence supporting first-line combination therapy with RTX and glucocorticoids for CAPS is limited, RTX was added in the current case with the aim of achieving earlier disease remission and reducing glucocorticoid exposure. Similar to our case, critical cases may benefit from the steroid-sparing ability of RTX and its prolonged effect on eliminating aPLs after the treatment. Although systemic glucocorticoid therapy is generally considered as the first-line treatment for idiopathic HES [26], in cases with secondary eosinophilia, treating the underlying disease is optimal [7, 26]. Our case was successfully treated with anticoagulation, glucocorticoids, PE, and RTX; as shown in our case, CAPS with PBE cannot be controlled by glucocorticoid monotherapy and can rapidly result in a devastating clinical course. Therefore, early diagnosis and initiation of aggressive therapy including anticoagulation, glucocorticoids, PE, and RTX is important. Since this report describes only a single case, further research is warranted to assess this therapy in patients with CAPS with PBE.

## Conclusion

We experienced a case with persistent fever, abdominal pain, skin lesions with pruritus, PBE, thrombocytopenia, multiple thromboses, and aPLs diagnosed with probable CAPS, which mimicked HES complicated by DIC at the time of initial assessment. Our case suggests that PBE can accompany CAPS and identifying aPLs is important for the diagnosis of CAPS when treating unexplained eosinophilia, thrombocytopenia, and thrombosis.

## Data Availability

All data are included within the article.

## Abbreviations

CAPS: Catastrophic antiphospholipid syndrome
APS: Antiphospholipid syndrome
aPLs: Antiphospholipid antibodies
PBE: Peripheral blood eosinophilia
HES: Hypereosinophilic syndromes
EGPA: Eosinophilic granulomatosis with polyangiitis
DIC: Disseminated intravascular coagulation
CT: Computed tomography
FISH: Fluorescence in situ hybridization
PSL: Prednisone
MPO-ANCA: Myeloperoxidase-antineutrophil cytoplasmic antibody
PR3-ANCA: Proteinase 3-antineutrophil cytoplasmic antibody
TTP: Thrombotic thrombocytopenic purpura
LA: Lupus anticoagulant
aCL: Anticardiolipin antibodies
aCL-β2GPI: Anticardiolipin β2-glycoprotein 1 complex antibodies
RTX: Rituximab
INR: International normalized ratio
IVIG: Intravenous immunoglobulin
